# Internal Lattice Reconfiguration for Diversity Tuning in Cellular Genetic Algorithms

**DOI:** 10.1371/journal.pone.0041279

**Published:** 2012-07-31

**Authors:** Alicia Morales-Reyes, Ahmet T. Erdogan

**Affiliations:** 1 School of Engineering, The University of Edinburgh, Edinburgh, Scotland, United Kingdom; 2 Computer Science Department, Instituto Nacional de Astrofisica, Optica y Electronica, Puebla, Mexico; University of Vermont, United States of America

## Abstract

Cellular Genetic Algorithms (cGAs) have attracted the attention of researchers due to their high performance, ease of implementation and massive parallelism. Maintaining an adequate balance between exploitative and explorative search is essential when studying evolutionary optimization techniques. In this respect, cGAs inherently possess a number of structural configuration parameters that are able to sustain diversity during evolution. In this study, the internal reconfiguration of the lattice is proposed to constantly or adaptively control the exploration-exploitation trade-off. Genetic operators are characterized in their simplest form since algorithmic performance is assessed on implemented reconfiguration mechanisms. Moreover, *internal* reconfiguration allows the adjacency of individuals to be maintained. Hence, any improvement in performance is only a consequence of topological changes. Two local selection methods presenting opposite selection pressures are used in order to evaluate the influence of the proposed techniques. Problems ranging from continuous to real world and combinatorial are tackled. Empirical results are supported statistically in terms of efficiency and efficacy.

## Introduction

Evolutionary Algorithms (EAs) are stochastic search techniques based on the principles of evolution. Amongst EAs, Genetic Algorithms (GAs) provide a competitive approach that has been successfully applied to a wide variety of difficult optimization problems [1]. Parallel Genetic Algorithms (PGAs) are classified by their grain in coarse/distributed and fine/cellular GAs [2], [3]. Coarse PGAs consist of several independent homogeneous or heterogeneous sub-populations evolving in parallel with individuals migrating among them. Fine or cellular GAs consist of a decentralized population where individuals interact with others located at nearby positions.

From the theoretical to the real world and particularly in the hardware implementation arena, GAs feasibility has been investigated. From the Very Large Scale Integration (VLSI) point of view, GAs possess important characteristics that makes them suitable for hardware implementation targeting real time applications. Parallelized versions of GAs have been used as optimizers and adapted for hardware implementation targeting real-time performance. Among problems solved by parallel GAs are: the image registration problem [4]–[6], the disc scheduling problem [7] and the GPS attitude parameters determination problem [8]–[11].

In [7], a cGA architecture was developed targeting the disc scheduling problem. It consists in finding the best attending tasks order to reduce the access time per request. In this architecture, a cellular GA encodes up to 32 queued requests ordered by a fitness function that minimizes latency and search time per request. The architecture achieved an scheduling time of up to 2 milliseconds per access request and 4 milliseconds of searching time per request. Thus, timing constraints were fulfilled. Ordered based crossover and mutation operations were performed. Ordered based crossover randomly selects chromosome positions in one parent, finds these positions in the second parent and copies corresponding alleles to the offspring. Thereafter, a reordering policy is applied, for selected genes to maintain their positions in the offspring. Ordered based mutation selects certain chromosome positions and mutate the alleles.

An image processing architecture was developed to tackle the image registration problem that presents real-time constraints [4], [5]. First, in [4] an architecture for image registration using a cellular GA was proposed. The image registration problem consists in matching a 2-D captured image to a reference image. A transformation between both images is necessary. The fitness function measures the number of corresponding pixels between captured and reference images. Once a perfect match has been found, the transformation encodes the object’s position and orientation. Each transformation parameters were encoded in 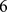 bits. At algorithmic level, an extra step was added to the canonical cGA procedure defined by Tomassini [12]. Each transformation parameter is incremented or decremented in one unit for promotion through hill climbing, while the best individual is always kept. Results showed that real-time constraints are fulfilled up to a tenth of a second to match the images, corresponding approximately to 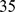 generations for 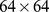 images.

In [5], an improved cGA architecture for image processing combining data compression and image registration is presented. For the image registration, a cGA determines an affine transformation for the image coordinates with respect to a reference image. The fitness function minimizes the error between the transformed and the referenced images. Then, the Discrete Cosine Transform (DCT) using a data compression algorithm is applied in order to reduce the memory storage requirements. In terms of speed, processing one chromosome took up to 2 milliseconds with limited accuracy for 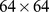 images.

A cellular GA architecture tackling the GPS attitude determination problem was developed by Xu et al. In [8], [9] a method to determine the attitude parameters of a vehicle based on the Global Positioning System (GPS) technology and the Ambiguity Function Method (AFM), is presented. The attitude parameters are determined by the vector difference between GPS receivers attached to the vehicle. Binary chromosome representation of 32 bits is used. The azimuth angle is encoded in the first 14 bits, the elevation angle in the next 10 bits and the base line length in the last 8 bits. In [9] a comparison among a panmictic GA, a panmictic GA with fine and coarse search stages, and a cellular GA with fine and coarse stages was carried out. Different array sizes were tested in order to find the best population size to solve the problem. Algorithmic performances were measured in terms of the average number of generations, and the hit rate together with the results accuracy.

Stefatos et al. extended the GPS attitude architecture presenting a novel approach from a fault tolerance perspective to deal with Single Hard Errors (SHEs) and implicitly with Single Event Upsets (SEUs), both are Single Event Effects (SEEs) subclasses [10]. Erroneous bit flipping in data registers, temporary (SEUs) or permanently (SHEs) is the effect of this kind of faults. If faulty registers correspond to critical data, the system’s functionality fails. In the proposed architecture, a faulty scenario considering stuck at zero faults at fitness score registers was targeted. In the cGA, individuals with faulty fitness scores are not selected and therefore their solution is not spread throughout the population.

The fault tolerant architecture is a double layer approach, each layer implements a cellular GA. A computational layer to determine the GPS attitude parameters and a control layer to determine the best configuration of individuals or Processor Elements (PEs) to overcome a faulty scenario. Several experiments were carried out, first only the computational layer was tested in order to observe how the cGA deals with the faults. Then the control layer was executed and a performance comparison is carried out. Results showed a significant improvement in the system’s performance when the control layer was active, in the worst case scenarios with 30% and 40% faulty PEs. Although, using the control layer allows an improvement in the system performance, there is a significant increment in the use of hardware resources. Another disadvantage is that the control layer was considered free of faults.

In [11] a high performance hardware architecture for the GPS attitude determination problem was presented; emphasis was paid to the speed, power consumption and hardware usage. Thus, a Coordinate Rotation Digital Computer (CORDIC) module was implemented to calculate the trigonometric functions required by the fitness function [13]. Results fulfil real-time constraints due to the simplified arithmetic units.

This article aims to provide a deeper insight into the great flexibility which cellular GAs inherently possess. When appropriately exploited, cGAs’ unique properties as regards the interaction of individuals enhance their searching abilities. In EAs, the main interest is to preserve population diversity through satisfactorily balancing the exploration and exploitation of solutions throughout the search space; in order to successfully converge to the global optimum in the minimum number of generations [14]. In this regard, structural properties of cGAs have shown their effectiveness in maintaining this balance, in several cases improving cGAs performance [15]. In this study, a dynamic cellular approach that *internally* reconfigures a traditional square topology into smaller sub-cellular structures is investigated.

Initial studies on Genetic Algorithms were mainly based on panmictic populations [2]. However, evident disposition to parallelization expanded GAs horizons, opening new areas for investigation, such as detailed by Cantú-Paz in his study on parallel GAs approaches in [16], [17]. In [16], parallel distributed GAs are analysed and new techniques are proposed to calculate population size according to problem’s difficulty. Moreover, migration is investigated as a crucial genetic operator and as a possible reason for PGAs speed-ups [18].

In order to validate the importance of decentralized GAs, authors compared their results to those of standard GAs, and frequently referred to modified or improved versions of standard GAs, such as steady state GA or generational GA. In several cases, cGAs were proved to perform better in terms of algorithmic efficiency and efficacy [19]. Moreover, the effect of synchronism in migration policies of distributed approaches using cGAs or panmictic independent populations, has been studied and compared. Asynchronous cGAs proved to be a competitive option that outperform synchronous ones, and in several cases also outperform panmictic distributed GAs in terms of real time [20]. On the other hand, simplicity in implementation of processor elements is also an advantage that has been investigated and applied [11].

Alba et al. have widely studied decentralized GAs [21]. An important area covered by their work is to compare distributed parallel GAs considering homogeneous or heterogeneous populations or a mixture of these. In [22], [23] a detailed analysis of several approaches at algorithmic and implementation levels is presented. Configurations of homogeneous sub-populations (only panmictic or cellular), heterogeneous sub-populations (a mixture of panmictic and cellular), comprising several migration and replacement criteria are evaluated. In general, their results showed the superiority of distributed cellular GAs in terms of efficacy over panmictic distributed PGAs. However, cellular GAs showed slower convergence times than standard steady-state distributed GAs. Communication among sub-populations was also assessed. Asynchronous communication leads to faster convergence and better speed-ups than synchronous ones.

In cellular GAs, the population is normally distributed in a torus like grid structure with wraparound edges. One individual is placed in each grid position and can only interact with nearby neighbours. Several parameters must be configured in order to reach the best algorithm performance, such as, shape and size of local neighbourhood and population topology, migration rate and frequency (in case explicit migration occurs), local selection and replacement policies, among others [24].

Initially, the study of cGAs has been developed following empirical knowledge through manually tuning and configuring the parameters mentioned above and through considering specific areas of application. However, there has also been an effort to provide a mathematical basis to explain their behaviour. For example, in order to provide a numerical relationship between grid structure and local neighbourhood, the dispersion of a points pattern has been defined as an appropriate measure known as the Neighbourhood to Grid Ratio (NGR) [15], [25], more details are provided in the next section. It has been demonstrated that by having different neighbourhood sizes and shapes in combination with, for example a square topology, different selection pressures are obtained. Selection pressure in cGAs is measured as the number of generations it takes for the best individual of an initial population to spread its solution throughout the grid. This measure is carried out by using only local selection. Shorter take-over times indicate high selection pressures and vice versa. A more detailed analysis will be carried out in the next section.

This article aims to investigate how selection pressure can be constantly or adaptively controlled in order to improve the performance of cellular GAs, as a result of the population topology and the local neighbourhood configuration. Previously, researchers have proposed dynamically reconfiguring the population topology [26]. However, a form of migration occurs during the relocation of individuals; this approach from now on is named as *external* lattice reconfiguration. Three main scenarios were evaluated: static (fixed topology shape), pre-programmed (reconfiguration at a predefined time slot) and adaptive (dynamic reconfiguration) criteria. Statistically significant results were obtained for several difficult benchmark problems. The mapping of individuals, after grid re-arrangement, induces a loss of the natural adjacency of individuals, due to toroidal connection. In this research, adjacency among individuals is maintained while constant or adaptive lattice reconfiguration is carried out by subdividing the entire population into smaller square, rectangular or linear toroidal arrays, with no induced migration among them apart from neighbourhoods overlapping. Proposed *internal* reconfiguration mechanisms are applied to a set of continuous, real and combinatorial problems covering characteristics such as multi-modality, epistasis, deceptiveness and non-regularity.

In the following section, a detailed explanation of the effect of controlling selection pressure through structural properties in cellular GAs is presented, together with proposed *internal* reconfiguration mechanisms.

## Methods

### Selection Pressure

Mechanisms proposed in this article control, constantly or adaptively, the induced selection pressure during evolution through the *internal* reconfiguration of the population topology. *Internal* lattice reconfiguration means that there is no alteration to the natural adjacency of individuals. Measuring selection pressure in cellular GAs has been fundamental and has allowed a more accurate study of their behaviour. Selection pressure in cGAs, is measured through the local selection of individuals, without intervention from other genetic operators; and it is empirically calculated as the number of generations the best individual of an initial population needs in order to spread its solution throughout the entire grid, concept also known as take-over time [12], [15].

Previously, Dorronsoro et al. proposed several constant and adaptive criteria to *externally* modify the induced selection pressure in order to improve the performance of cGAs [26]. However, their approach considers an inherent migration mechanism; through it, new positions are calculated for individuals when the population topology is changed among square, rectangular and narrow lattice shapes. Although results showed an improvement in performance for several benchmark problems, it is not clear if this improvement is purely due to controlling the NG ratio or if it is also a consequence of inherent migration. In this article, the proposal is to internally reconfigure the population’s topology, constantly or adaptively, while maintaining the original adjacency among individuals.

Selection pressure in cGAs provides important information about the evolutionary process showing levels of exploration or exploitation that certain population topology contribute to the search. In [26], diversity changes are measured as speed variations of the average population fitness score or the population entropy. If the average fitness increases drastically from one generation to the other, the search process proceeds rapidly and therefore exploitation is high. On the other hand, if the average fitness of the population remains the same or has slightly changed in comparison to previous generations, the population is slowly evolving and therefore individuals are widely spread throughout far-off regions of the search space. Although, this analysis covers several possible scenarios, it does not include all of them. For example, individuals can be loosely located over a multi-modal landscape and the average fitness could be rapidly increasing or vice versa.

Researchers have demonstrated that the exploration-exploitation trade-off can be implicitly controlled in cGAs through the configuration of the population topology and the neighbourhood [27], [28]. A measure known as the neighbourhood-grid ratio (NGR) has been defined in order to describe its behaviour. The NGR was first introduced by [15]. Its calculation corresponds to measuring the dispersion of 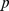 points (each individual position) centred at 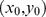. A dispersion measure is used because other possible measures, such as the radius of a circle, would give the same value for different population topologies. The dispersion of a points pattern is calculated as follows:


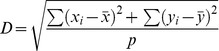
(1)

where 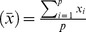 and 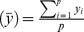. Hence, the NGR is given by


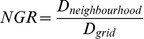
(2)

**Figure 1:**
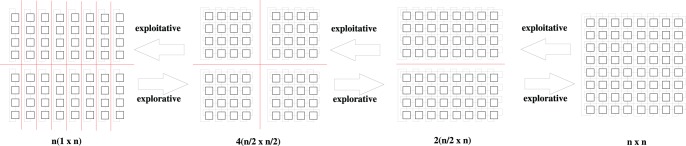
Neighbourhood-grid ratios corresponding to cellular structures and sub-structures. Each topology configuration induces a different level of selective pressure. Applying constant or adaptive *internal* reconfiguration criteria globally affects the search making it more explorative or exploitative while maintaining adjacency among individuals.

In Figure 1, four population configurations are drawn. Internally, smaller cellular structures imply a higher or lower NGR, that as a whole would modify the search making it more exploitative or explorative. A 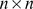 configuration, where 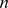 is the number of individuals per grid side, is the only topology which NGR directly represents its level of selective pressure. A 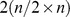 configuration divides a square grid in two rectangular topologies with 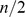 individuals on the shorter side. A 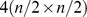 configuration divides a fully connected square topology in four smaller square toroidal grid configurations with 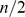 individuals per side. Finally, linear toroidal arrays are formed with length of 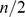 individuals. Configurations denoted by 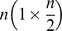 and 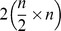 are implemented in both vertical and horizontal alignments. These lattice configurations have been used in the experimental set-up. In order to assess these internally different configuration topologies in terms of selective pressure, an empirical study of the take-over times is necessary.

For most topologies in Figure 1, a compact Von Neumann local neighbourhood is used consisting of individuals located at North, East, South and West with a Manhattan distance of one from the central individual; except for linear lattices (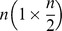) that employ a linear local neighbourhood with a Manhattan distance of two. As an example, for a population of 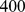 individuals, dispersion measures are 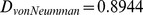 with local Von Neumman neighbourhood and 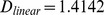 for the linear case. If individuals are distributed on a 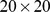 square topology, corresponding NG ratios, as regards each independent cellular structure, are as follows:


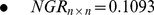



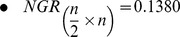



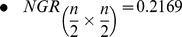



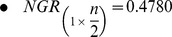


Different ratios means having higher or lower selection pressures. Therefore, decentralized GAs are structurally capable of modifying selective pressure while changing the population’s structural configuration. Lower ratios means more exploration is performed while higher ratios imply more exploitation. However, the NGR is a representation of the selective pressure in a fully connected structure or sub-structure. Therefore, it does not reflect the overall induced selective pressure in topologies configurations formed by several cellular sub-structures, like those shown in Figure 1. In order to analyse the levels of selective pressure that each topology configuration induces, an experimental assessment is carried out to calculate the take-over times following a constant *internal* lattice reconfiguration criterion.

Take-over time or proportional growth of the best individual reflects how long it takes for the best individual to spread its solution throughout the whole lattice, applying only local selection. Thus, longer take-over times represent lower selection pressure and therefore more explorative behaviour. In contrast, shorter take-over times correspond to higher selection pressure, equivalent to a more exploitative search.

At a local level, two different selection methods are applied; widely known binary tournament selection and, ad-hoc for cellular GAs, anisotropic selection proposed by [29]. These selection methods provide distinctively opposite selection pressures. Binary tournament is highly exploitative in comparison to anisotropic selection which is more explorative.

**Figure 2:**
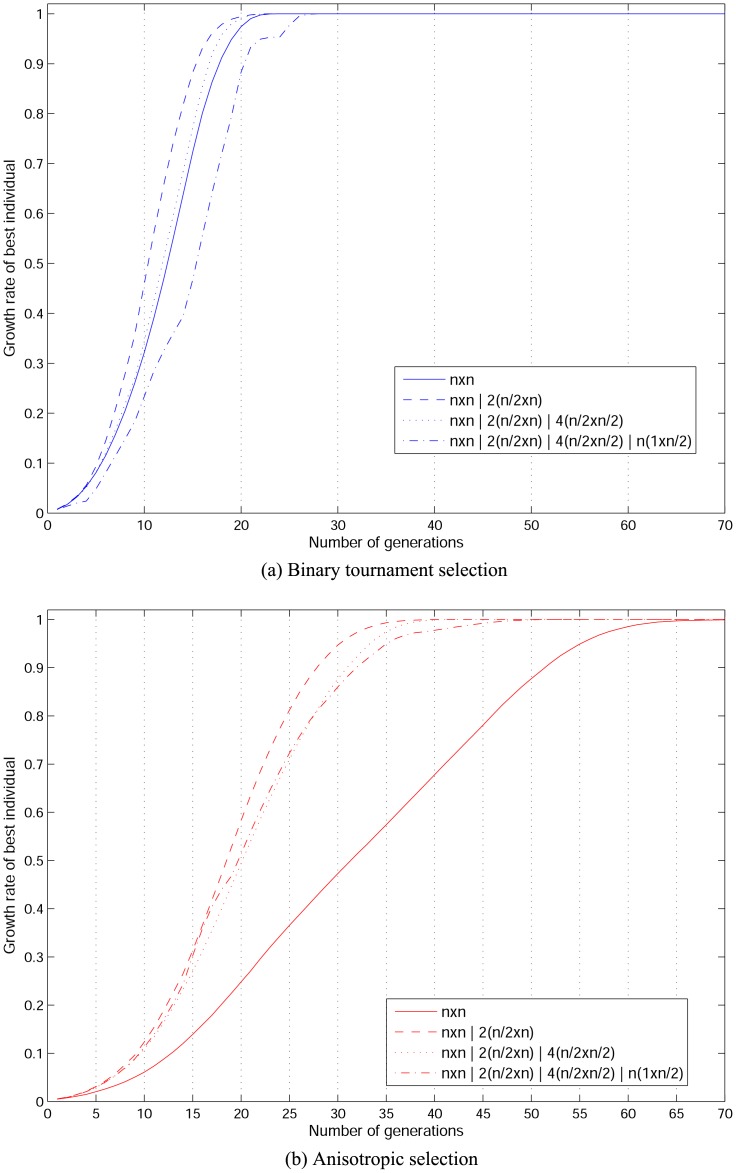
Take-over times for constant lattice reconfiguration locally applying binary tournament or anisotropic selection. The growth rate indicates the average number of generations for a hundred experiments that takes the best individual to spread its solution throughout the grid.

In Figure 2, take-over times for both selection methods are shown. A hundred experiments with a population of 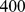 individuals, placed on a 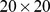 grid configuration, were performed. Only constant lattice reconfiguration between a square lattice and topologies drawn in Figure 1 is performed. Constant reconfiguration occurs every 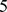 generations. For 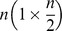 and 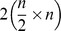 formations, horizontal or vertical alignments are executed with probability 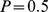. Adaptive *internal* lattice reconfiguration is not subject to take-over time analysis since it depends on phenotypic or genotypic diversity; information not available when applying only local selection to a single individual. On the other hand, due to the stopping criterion which evaluates the average fitness score for the whole population, and in order to assess selective pressure on an internally different grid configuration; reconfiguration occurs between a fully connected 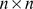 topology and one of the other configuration options.

For both local selection methods in Figure 2, solid lines correspond to selective pressure on a fully connected 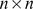 square topology. On a square topology, binary tournament presents higher selection pressure in comparison to anisotropic selection which is more explorative. Once constant reconfiguration is applied, anisotropic selection becomes more exploitative when either internal configuration is applied. This behaviour is not repeated through binary tournament selection, presenting a slightly more explorative behaviour when reconfiguration occurs between a square and a 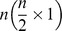 topology. However, in both selection cases when reconfiguration happens between 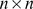 and 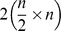 topologies, the highest selection pressure is induced. Specific constraints based on take-over time analysis are considered in the proposed algorithmic approach.

The main difference between *internal* (proposed in this article) and *external* [26] lattice reconfiguration approaches is that the former maintains individuals’ adjacency while the later induces a form of explicit migration which is an influential genetic operation to restore diversity during the evolutionary process. One of the main aims in this article is to show the effect of solely manipulating the population’s topology as a way to control diversity without taking advantage of other genetic operations. To show the effect of having the added effect of explicit migration, an experimental assessment on genotypic and phenotypic entropy to solve the ECC problem (see Section) is carried out. Lattice reconfiguration occurs once at a predefined time slot which is an average half the number of generations that takes for a cGA on a square topology to find the problem’s solution. Both reconfiguration mechanisms are assessed locally applying anisotropic selection which showed a wider span of selective pressure in contrast to binary tournament selection. For *external* lattice reconfiguration, evolution starts on a square topology and continue on a rectangular grid, making the search going from more exploitation to more exploration. On the other hand, for *internal* reconfiguration, initially the population evolves on an *internal* arrangement of four square cellular sub-structures (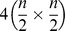) and lattice is then reconfigured to a square topology. For the *internal* mechanism, this means going from a more exploitative to a more explorative behaviour as shown by the take-over time experimental analysis when constant lattice reconfiguration takes place.

**Figure 3:**
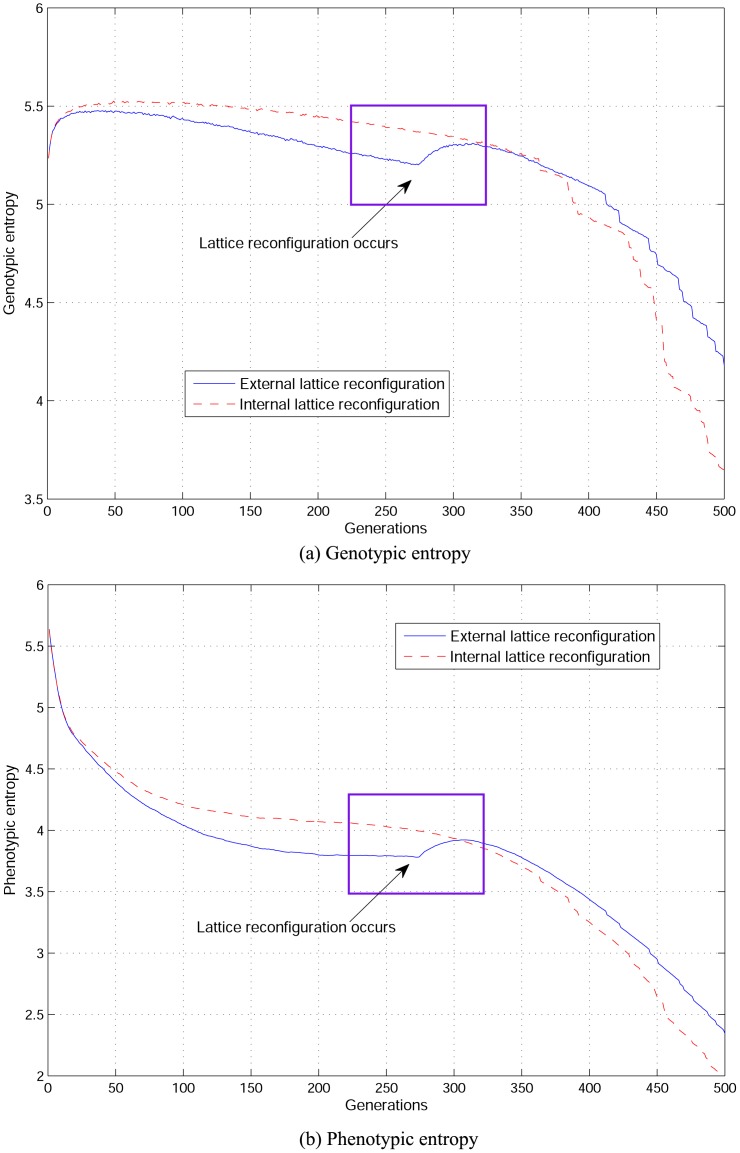
Comparing genotypic and phenotypic entropy for *internal* and *external* lattice reconfiguration.

Entropy results are obtained from a hundred experimental samples, see Figure 3. Dashed lines correspond to *internal* reconfiguration and solid lines to *external* reconfiguration. A square shows the moment both *internal* and *external* reconfiguration is performed. For both entropy measures, change in diversity is clear when *external* reconfiguration occurs; as a result of grid change and individuals’ migration. In contrast, after reconfiguration is internally performed, entropy measures do not change abruptly as a consequence of maintaining individuals’ adjacency.

Another observation from entropy results in Figure 3 is that entropy measures are slightly higher for *internal* lattice reconfiguration during the initial stages of evolution and until reconfiguration occurs. However, in this experimental assessment reconfiguration is applied only once while the algorithmic approach and the experimental set-up are based on the take-over times analysis which consists in systematic reconfiguration among all proposed internal topology configurations. In the following section, details of the algorithmic procedure and diversity measures for lattice reconfiguration are provided.

### Cellular Configuration

This study proposes to constantly or adaptively reconfigure the internal population topology to modify the induced selective pressure, and thus achieve an appropriate balance between searching exploration and exploitation, essential for maintaining population’s diversity during evolution. In this proposal, lattice reconfiguration is implemented by subdividing a squared population into smaller squared, rectangular or linear toroidal arrays. A similar approach has been referred to as a parallel cellular GA [21]. However, that approach has a fixed *internal* configuration. Whilst an homogeneous distributed or an island model cGA is one possible perspective [30], migration policies have not been investigated nor implemented. During constant or adaptive periods of evolution, smaller topologies maintain internally toroidal connections [31], [32].

**Figure 4:**
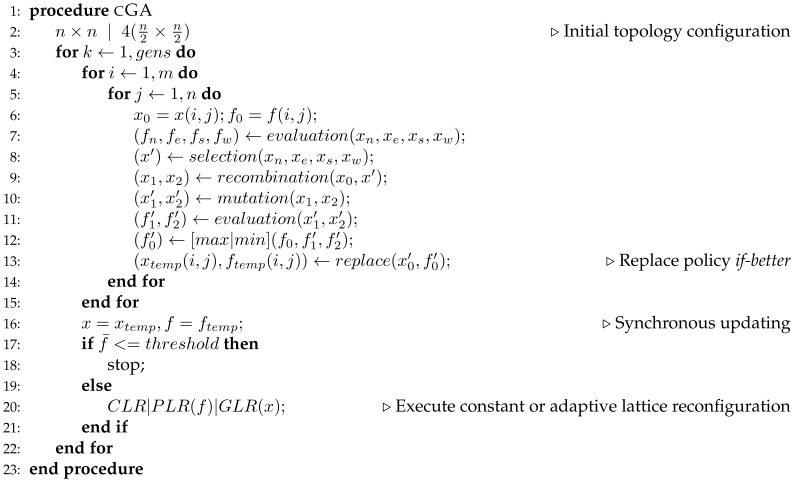
Pseudocode for the proposed reconfigurable cellular GA.

In Figure 4, a pseudocode for the proposed cellular GA approach is included. A single random seed is used to generate the entire initial population, two topology configurations are set to evolve the first generations: a 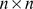 or a 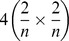 lattice. For each individual, neighbours located at North, East, South and West positions are evaluated in order to select a second parent for reproduction. Two local selection methods, presenting opposite selection pressures, are applied for experimental purposes. Binary tournament presents higher selection pressure than anisotropic selection.

Binary tournament randomly chooses two individuals from the local neighbourhood and the best one (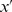) is mated with the central individual (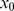). On the other hand, anisotropic selection requires an extra parameter (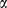) that leads the search in North-South or East-West grid directions. Thus, anisotropic probabilistic equations are defined for each individual in the neighbourhood as: 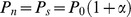 and 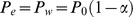; where 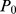 is the uniform probability for each neighbour to be selected. If 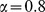 and 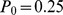, individuals at North and South positions are assigned with higher probabilities for selection compared to East and West individuals that remain with lower selection probabilities. In this way the search process is directionally guided and adjusting the 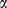 parameter would supply higher or lower selection pressure. For experimental purposes, 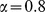 has been used due to presenting lower selection pressure and thus being more explorative. Moreover, if 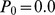 all neighbours have same selection probability and anisotropic selection behaves as binary tournament selection. Although fewer studies have been reported using this local selection method in cGAs, anisotropic selection does provide more flexibility in terms of selective pressure, making use of cGAs’ structural properties, and even more when the population topology is reconfigured; this being the main perspective in this research [29], [33].

Recombination is performed using Single Point Crossover (SPC) with highest probability and constant mutation probability. Both genetic operators have been applied in their simplest form, as it is not the objective of this research to evaluate the effect of either of them but to widely investigate the effect of dynamically changing selection pressure through cGAs structural properties. Although it is outwith the scope of this paper, particular attention should be paid to mutation as its effect has been modelled for cellular GAs as an essential control parameter to lead or mislead the search [34], [35]. Replacement takes place following an *if-better* policy. Only if.

In Figure 4, line 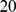, 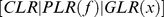 corresponds to constant lattice reconfiguration, phenotypic lattice reconfiguration or genotypic lattice reconfiguration, respectively.

CLR consists in constant cyclic reconfiguration among internal lattice topologies shown in Figure 1. Every 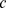 generations the topology is reconfigured to a different topology configuration following a cyclic pattern. For rectangular (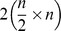) and linear (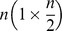) grid configurations, both horizontal and vertical alignments are performed with 50% probability. The overall induced selective pressure varies according to the take-over time analysis presented in previous section.

Grid reconfiguration based on phenotypic diversity (PLR procedure) evaluates differences in phenotypic entropy, 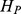, among consecutive generations:


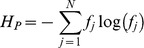
(3)

where 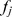 is the proportion of individuals in one generation having fitness 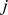. Thus, the difference between 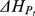, 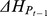 and 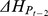 would determine if phenotypic diversity in current generation has or has not significantly changed with respect to previous generations; where 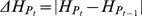.

**Figure 5:**
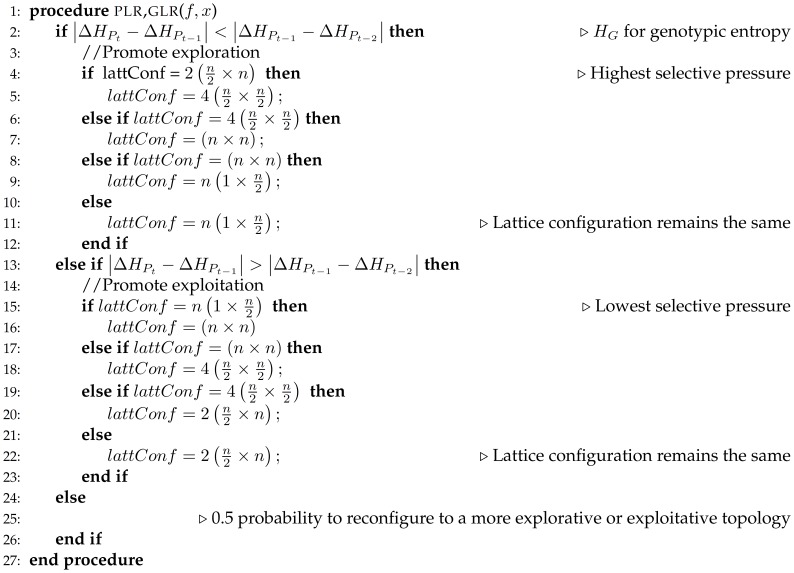
Pseudocode for phenotypic or genotypic lattice reconfiguration through binary tournament selection.

**Figure 6:**
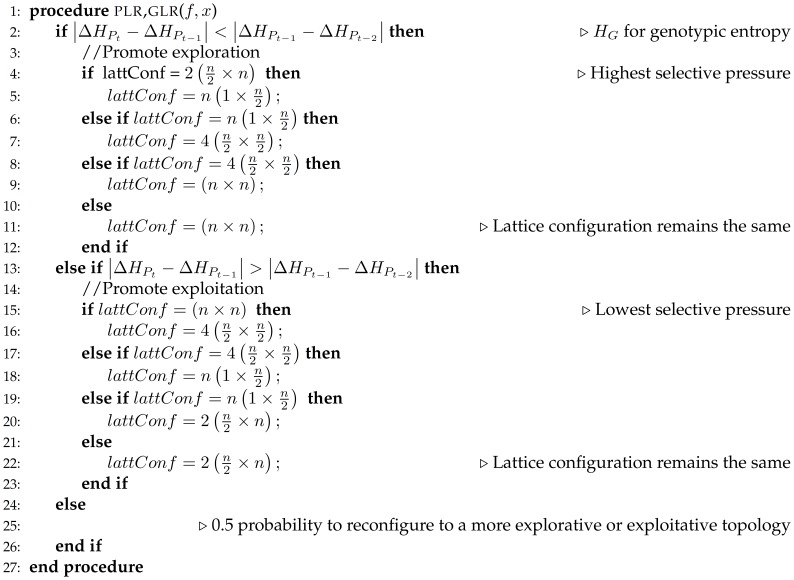
Pseudocode for phenotypic or genotypic lattice reconfiguration through anisotropic selection.

In Figures 5 and 6, adaptive reconfiguration mechanisms for binary tournament and anisotropic local selection methods are described. If diversity among phenotypes decreases, 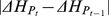 is less than 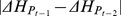 then exploration should be encouraged by switching to a lattice configuration that presents in average lower selective pressure. If the same condition indicates an increase on phenotypic diversity, in order to make the search more aggressive, exploitation should be promoted by increasing selection pressure. Otherwise, population’s topology remains the same.

On the other hand, determining genotypic diversity requires to calculate the Hamming distance among chromosomes. The distance between current (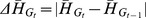) and previous (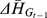, 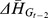) generations is calculated in order to assess genotypic diversity changes. In a similar way to phenotypic diversity, if 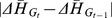 difference is less than 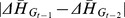, exploration should be encouraged through a lattice configuration which induces a lower selective pressure. On contrast, exploitation should be promoted by increasing overall selection pressure.

For both diversity metrics, if the change rate of phenotypic or genotypic diversity equals zero, it means the search process has reached a plateau and no more competitive solutions would arise. Therefore, the effect of reconfiguring the grid will not significantly affect the evolutionary process. Because the proposed adaptive criteria do not evaluate the growing or decaying direction of diversity, the algorithmic approach follows a 0.5 probability policy to reconfigure the grid to an internal configuration that provides either a more exploitative or more explorative behaviour.

In [26], conditions for lattice *external* reconfiguration imply the relocation of individuals to new positions, inducing a kind of migration among them. Hence, the impact of reconfiguring the grid is not completely clear; as migration is an influential operation that can by itself change significantly the performance of cGAs [18], [19]. Reconfiguration mechanisms proposed here, do not induce any explicit migration and once topology configuration has *internally* reconfigured, a toroidal connection with wraparound edges is maintained in all cellular sub-structures. Thus, higher or lower intensities of selective pressure are applied throughout the landscape after lattice reconfiguration takes place while individuals’ adjacency is maintained.

Pre-programmed criterion by Dorronsoro et. al. consists in *externally* changing topology’s shape after a certain number of generations, that is half way through a successful run for a square topology. For the adaptive approach, authors determined a constant parameter as a threshold to measure phenotypic and genotypic diversity and switch among topology configurations [26]. Criteria herein proposed avoid the use of an extra parameter and directly detect significant changes while extending the generation-range to measure diversity. In the following section, results analysis including the experimental set-up is presented.

## Results

### Experimental Set-up

In order to assess the dynamic reconfiguration criteria proposed in this article and as an initial comparison, pre-programmed and adaptive criteria of Dorronsoro et al. have been applied to the benchmark problems tackled in this research [26].

A comparison between centralized versus decentralized GAs is outwith the scope of this article. However, several authors have demonstrated in previous research that cellular GAs outperform serial GAs when solving difficult optimization tasks [8], [12], [21]. In this article, as a reference point, a traditional toroidal square topology is used for performance comparison.

### Benchmark Problems

In Table 1, a summary of mathematical equations and properties of the benchmark problems is included. Three continuous problems: the Rastrigin, the Griewank and the Langerman functions are assessed [36], [37]. Three real-world problems are tackled: the Frequency Modulation Sound (FMS) problem, the System of Linear Equations (SLE) problem [21] and the GPS attitude determination problem which has been previously tackled by the authors in [38]. Finally, three combinatorial problems are evaluated: the Massively Multi-modal Deceptive Problem (MMDP), the Minimum Tardy Task Problem (MTTP), the P-Peaks problem [39] and the Error Correcting Code (ECC) design problem [21]. Chromosome encoding for all problems is binary. The stop condition is defined by the average population’s fitness (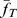) score for each problem.

### Experimental Constraints

Crossover and mutation operators are configured in a simple form and are kept constant during the search process. Single Point Crossover (SPC) operates pairs formed by a central individual and an individual selected from the local neighbourhood with probability 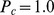. SPC randomly selects a single point for recombination. On the other hand, mutation is performed after recombination with probability 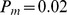. Neither genetic operators are specifically evaluated in this article and are configured in their simplest form.

Replacement policies also play an important role in GAs. In cGAs, an offspring can replace a current individual in the following three ways: 1) always, 2) only if it has a better fitness score or 3) following some other condition. In this research an *only replace if better* policy is followed. On the other hand, synchronous population updating is also considered. Synchronous updating requires current and next generations to be stored separately; once all individuals have evolved, new offspring will entirely replace the current population. Asynchronous updating has also been investigated and in several problems outperforms synchronous updating in terms of convergence time although the accuracy of the results and their hit rate are negatively affected [20].

The following experimental constraints are evaluated:

- A population size of 400 individuals was used for most problems. Due to their size, GPS and MTTP problems are tackled using a population size of 64 and 100 individuals respectively.
- Binary encoding is used in all problems. In continuous and real problems, each variable is encoded in 10 bits, except for the GPS attitude problem where each variable is encoded in 14, 10, 8 bits respectively. Binary encoding is implicit in combinatorial problems.
- Local neighbourhood configuration is Von Neumann or linear composed by four individuals plus a central individual.
- One hundred independent runs are carried out per experimental reconfiguration criterion.
- A problem specific threshold based on population average fitness is used as a stop condition.
- A limit of 500 generations is used in most problems, except for the Langerman function, the SLE and the MMDP problems with a limit of 700 and the ECC problem with 2500 generations.

The same reconfiguration mechanisms are applied to all problems and results are analysed based on convergence time and hit rate. The accuracy of results is determined by problem specific thresholds, see Table 1. The stop condition evaluates population’s average fitness score. An accepted stop condition considers that experimental samples should evolve until a solution of the same quality is found and not after a certain number of generations [20]. The stop condition herein considered has been defined due to previous research constraints related to fault tolerance. Although fault tolerance is a different topic, outwith the scope of this article, the decision was made to keep the same stop condition for consistency. Results herein obtained will be taken up again for future research in the area of fault tolerance. For further reference readers can consult [38], [40].

**Table 1:** Benchmark problems.

Problem	Fitness function	Properties
Rastrigin	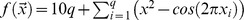	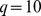 , 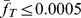 , multi-modal, regular
Griewank	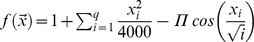	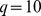 , 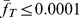 , multi-modal, epistatic, regular
Langerman	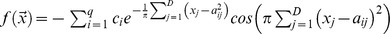	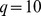 , 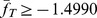 , multimodal, epistatic, non-regular
FMS	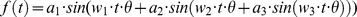	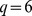 , 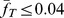 , multimodal, epistatic, non-regular
SLE	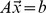	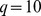 , 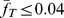 , epistatic
GPS	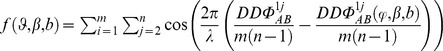	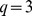 , 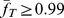 , multimodal, epistatic, non-regular
MMDP	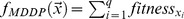	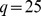 , 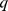 sub-problems, 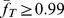 , multi-modal, combinatorial
MTTP	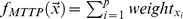	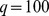 , 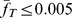 , epistatic, NP-combinatorial
P-Peaks	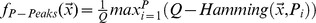	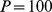 peaks, 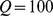 bits, 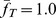 , non-regular, non-manually tunable
ECC	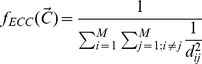	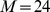 codewords, 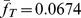 , non-regular, non-manually tunable

### Statistical Tests

In order to statistically support results so far obtained, the Lilliefors normality test, at 5% of significance, is performed on each set of convergence time results. This test is suitable when a fully-specified null distribution is unknown, in contrast to the Kolmorov-Smirnov test. Once normality of results has been established, an Analysis of Variance (ANOVA) is applied among results that follow a normal distribution whereas the Kruskal-Wallis test is applied to results that are not normally distributed. For sets of results with non-normal distributions, dispersion of data is calculated using the *mean absolute deviation* and *italics* are used to highlight these cases in the results tables in the next section.

In the next section, results are summarized in two tables, one for static, pre-programmed and constant reconfiguration criteria and a second one for adaptive approaches. Then, in order to prove that a certain approach outperforms others, in terms of convergence time, a multiple comparison test is carried out, after a statistical analysis is done independently for each table of results. In contrast, if statistical proof is obtained only in one of the two tables, a multiple comparison test is applied separately.

In the results tables, where a statistically significant difference at 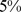 has been found among convergence time results, this has been represented by the symbol 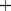. On the contrary, a 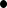 symbol represents results which are not statistically different, and where therefore the application of proposed dynamic criteria makes no difference in terms of the number of generations. On the other hand, having statistically different results does not mean the proposed approaches definitively improve cGAs performance. This statistical analysis only evaluates the efficiency of proposed techniques. Thus, the efficacy of results has also been statistically considered.

The hit rate has been statistically evaluated as a Bernoulli trial. A random experiment whose result is either success or failure can be considered a Bernoulli trial [12]. For each dynamic lattice reconfiguration criterion, 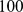 experiments were carried out. In successful experiments, the global optima, at a certain threshold, is reached. Measuring the standard deviation of successful experiments percentages provides a numerical value that indicates how significantly different are these success search rates. The standard deviation for each experimental sample is calculated as follows:


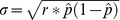
(4)

where 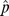 represents the probability of successful experiments and 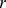 is the total number of experiments. In results tables, the standard deviation is shown next to hit rates percentages for each experimental sample. In the next section, results are detailed.

### Results Analysis

Results analysis aims to empirically support the main hypothesis of this study: dynamic modification of the internal configuration of the population’s topology would lead to a significant improvement in cGAs’ performance. At a local level, as explained previously in Section, two different local selection methods have been applied. The main difference between them consists of inducing higher (binary tournament) or lower (anisotropic) selection pressure. These two methods were chosen because of their considerably different selection intensity. Thus, it is possible to separately evaluate 1) the effect of having a highly exploitative or explorative cellular GA, due to local selection, and 2) the added flexibility of dynamically reconfiguring the population topology in order to supply the search process with a more convenient and balanced exploitation-exploration trade-off.

For presentation, results are divided by local selection method. Two tables of results are organized: the first includes static, pre-programmed (*external*) reconfiguration and constant (*internal*) reconfiguration. The second group presents adaptive approaches for both *external* and *internal* lattice reconfiguration approaches.

In the tables, numbers in **bold** indicate the best average convergence time and *italics* are used to distinguish between standard deviation (normal distributions) and mean absolute deviation (non normal distributions) as dispersion measures. Next to the hit rates, corresponding Bernoulli trial standard deviations are shown in small numbers.

### Binary Tournament Local Selection

Tables 2 and 3 present results for static, pre-programmed, constant and adaptive lattice reconfiguration criteria through binary local selection. The Rastrigin function hit rate is 100% for all configurations, and convergence time is slightly affected through lattice reconfiguration. Statistical proof is obtained for the two best criteria: square 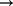 constant and 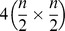 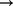 adaptive phenotypic *internal* reconfiguration. On the other hand, adaptive approaches do not show significant difference in convergence time.

**Table 2:** Convergence time and hit rate results for continuous, FMS and SLE problems through static, preprogrammed (*external*) and constant (*internal*) lattice reconfiguration with *binary tournament local selection*.

Static/Preprog.					
*External* Reconfig.	Rastrigin	Griewank	Langerman	FMS	SLE
Square	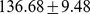	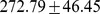	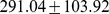	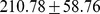	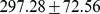
	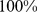	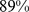 , 3.12	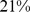 , 4.07	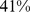 , 4.91	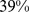 , 4.87
Rectangular	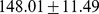	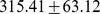	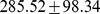	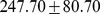	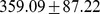
	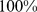	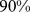 , 3	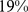 , 3.92	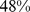 , 4.99	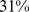 , 4.62
Narrow					
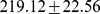	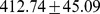	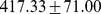	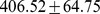	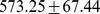	
	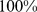	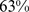 , 4.82	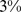 , 1.70	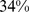 , 4.73	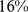 , 3.66
Square-Narrow					
	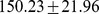	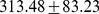	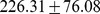	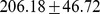	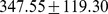
	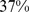 , 4.82	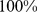	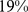 , 4.20	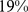 , 3.92	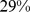 , 4.53
Narrow-Square	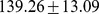	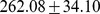	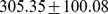	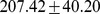	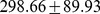
	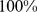	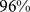 , 1.95	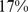 , 3.75	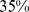 , 4.76	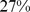 , 4.43

**Table 3:** Convergence time and hit rate results for continuous, FMS and SLE problems through *external* and *internal* adaptive lattice reconfiguration with *binary tournament local selection.*

Adaptive Phenotypic					
*External* Reconfig.	Rastrigin	Griewank	Langerman	FMS	SLE
Rectangular 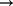 Adapt.					
	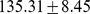	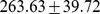	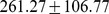	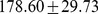	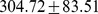
	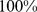	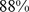 , 3.24	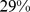 , 4.53	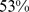 , 4.99	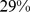 , 4.53
Narrow 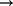 Adapt.	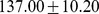	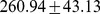	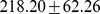	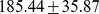	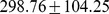
	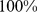	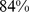 , 3.66	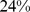 , 4.27	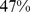 , 4.99	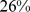 , 4.33
*Internal* Reconfig.					
Square 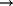 Adapt.	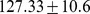	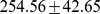	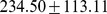	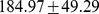	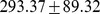
	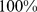	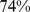 ,4.38	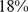 ,3.84	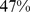 ,4.99	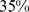 ,4.76
4( 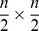 ) 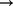 Adapt.	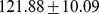	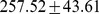	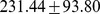	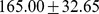	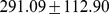
	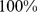	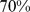 , 4.58	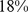 , 3.84	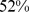 , 4.99	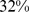 , 4.66

Similar results in the number of generations are obtained for the Griewank function, statistical difference at 5% is proved for square 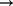 constant *internal* reconfiguration with respect to static and pre-programmed criteria. However, in terms of success search rate, pre-programmed square 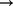 narrow approach shows major advantage in this regard, from 80% (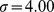) to 100% 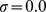, based on calculated sample standard deviations. On the other hand, for adaptive approaches the best convergence time is obtained by square 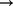 adaptive genotypic *internal* reconfiguration with statistical difference. The best hit rate is 88% (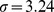) for rectangular 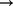 adaptive *external* reconfiguration, which is not as good as the pre-programmed approach.

The Langerman function is the most difficult in the continuous set of problems tackled in this study; hit rates are not higher than 29%. The improvement in results for lattice reconfiguration approaches is not significant, either in convergence time or in the hit rate for all proposed criteria through binary tournament local selection. Similar results were obtained for the FMS problem. No significant improvement in terms of convergence time was obtained through any of the proposed approaches. Standard deviations for hit rates do not show significant statistical difference either.

From these two sets of results, it is worth noticing that the best convergence times in Table 2 correspond to constant approaches. However, hit rates in some cases are higher for either static or pre-programmed criteria, with statistical proof, for example, in the Griewank function. For adaptive approaches, in Table 3, best convergence times correspond to *external* reconfiguration techniques, however these results are not statistically supported. Results consistency for convergence times is maintained for most problems, except in the Langerman function. In terms of convergence time, the SLE problem presents a significant difference for square 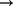 constant *internal* reconfiguration. However, in terms of hit rate, traditional static square grid provides the highest efficacy (39%). In contrast, the best adaptive square 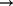 adaptive and 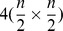 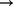 adaptive *internal* phenotypic and genotypic based criteria reach a hit rate of 35%, with no meaningful difference due to 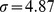 and 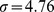 respectively. The convergence times for adaptive criteria are not different, and the consistency of the results is not well maintained. Multiple comparison tests are not carried out for results in Tables 2 and 3 due to the lack of statistical difference in convergence time for most of the problems.

In Tables 4 and 5, corresponding results for the GPS and combinatorial problems are shown with binary tournament selection. For the GPS attitude determination problem, through binary local selection, no statistical proof was obtained in terms of convergence time and hit rate for both sets of results. In contrast, the MMDP statistical proof was found in convergence time for 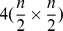 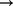 constant *internal* reconfiguration. However, the best hit rate of 92% (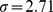) is reached through pre-programmed narrow 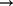 square *external* reconfiguration, in comparison to 87% (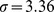) of former approach. However, the narrow 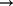 adaptive genotypic based approach, *external* reconfiguration, provides best efficiency and efficacy.

**Table 4:** Convergence time and hit rate results for GPS and combinatorial problems through static, preprogrammed (*external*) and constant (*internal*) lattice reconfiguration with *binary tournament local selection*.

Static/Preprog.					
*External* Reconfig.	GPS	MMDP	MTTP	P-Peaks	ECC
Square	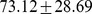	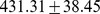	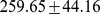	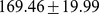	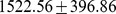
	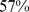 , 4.95	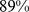 , 3.12	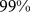 , 0.99	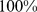 ,	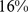 , 3.66
Rectangular	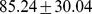	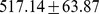	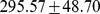	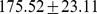	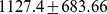
	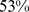 , 4.99	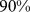 , 3.00	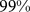 , 0.99	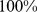 ,	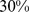 , 4.58
Narrow	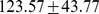	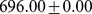	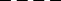	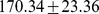	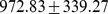
	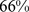 , 4.73	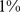 , 0.99	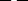	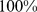 ,	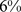 , 2.37
Square-Narrow	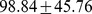	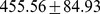	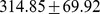	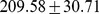	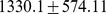
	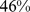 , 4.98	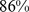 , 3.46	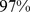 , 1.70	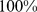 ,	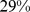 4.53
Narrow-Square	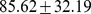	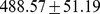	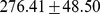	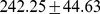	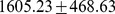
	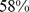 , 4.93	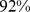 , 2.71	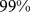 , 0.99	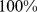 ,	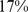 , 3.75

**Table 5:** Convergence time and hit rate results for GPS and combinatorial problems through *external* and *internal* adaptive lattice reconfiguration with *binary tournament local selection*.

Adaptive Phenotypic					
*External* Reconfig.	GPS	MMDP	MTTP	P-Peaks	ECC
Rectangular 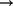 Adapt.	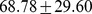	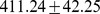	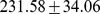	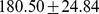	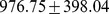
	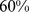 , 4.89	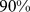 , 3.00	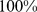 ,	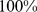 ,	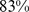 , 3.75
Narrow 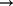 Adapt.	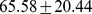	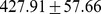	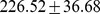	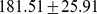	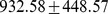
	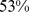 , 4.99	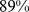 , 3.12	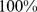 ,	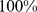 ,	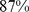 , 3.36
*Internal* Reconfig.					
Square 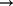 Adapt.	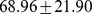	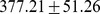	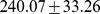	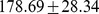	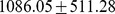
	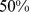 , 5.0	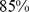 , 3.57	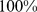 ,	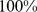 ,	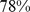 ,
4( 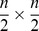 ) 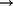 Adapt.	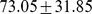	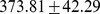	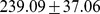	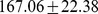	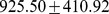
	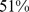 , 4.94	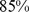 , 3.57	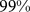 , 0.99	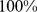 ,	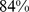 , 3.66

For the MTTP, high hit rates are in general obtained. There is a statistical difference for the best configuration case among static, pre-programmed and constant approaches: square 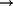 constant *internal* reconfiguration. Similarly for adaptive approaches, statistical difference is proved for the best adaptive mechanism: narrow 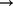 adaptive genotypic based *external* reconfiguration. After a multiple comparison test is performed for both sets of results, no statistical proof was found for the adaptive approach in terms of convergence time. The P-Peaks problem through binary tournament selection presents no statistical difference for either static, pre-programmed, constant or for adaptive approaches in terms of convergence time, and hit rates were in all cases 100%.

The ECC problem presents statistical difference in terms of convergence time in both Tables 4 and 5. The best performance is achieved by genotypic based *external* reconfiguration when evolution starts on a rectangular configuration.

At this stage, an interesting preliminary conclusion indicates that the difference in performance between constant and adaptive approaches is not as substantial as expected. Moreover, in Table 4, the best results, in terms of convergence time, correspond to constant *internal* reconfiguration. However, better efficacy is obtained through static and pre-programmed *external* reconfiguration criteria for SLE, GPS and MMDP problems. Another interesting observation in Table 5, for all problems, is that best convergence times are obtained through adaptive genotypic based *external* reconfiguration.

In general, binary tournament local selection provides a narrow span for selective pressure when internally reconfiguring the grid, in comparison to anisotropic local selection, see Figure 2. However, several advantages in performance have been achieved for some problems with statistical proof.

### Anisotropic Local Selection

Results obtained through anisotropic local selection are presented in this subsection. In Tables 6 and 7 results for continuous problems and the FMS problem are presented. Anisotropic local selection with constant 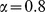 presents a more explorative behaviour if compared to binary tournament selection. Moreover, this selection method also makes use of structural properties in cGAs, due to neighbourhood configuration in which the location of individuals for selection is implied.

**Table 6:** Convergence time and hit rate results for continuous, FMS and SLE problems through static, preprogrammed (*external*) and constant (*internal*) lattice reconfiguration with *anisotropic local selection*.

Static/Preprog.					
*External* Reconfig.	Rastrigin	Griewank	Langerman	FMS	SLE
Square	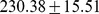	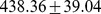	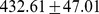	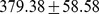	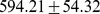
	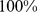	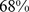 , 4.66	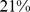 , 4.07	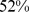 , 4.99	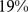 , 3.92
Rectangular	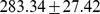	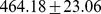	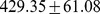	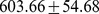	
	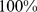	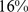 , 3.66	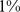 , 0.99	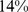 , 3.46	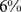 , 2.37
Narrow	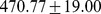	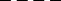	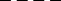	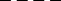	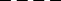
	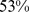	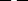	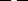	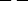	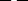
Square-Narrow	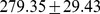	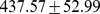	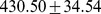	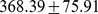	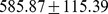
	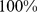	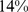 , 3.46	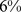 , 2.37	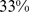 , 4.70	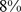 , 2.71
Narrow-Square	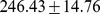	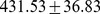	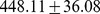	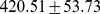	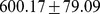
	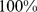	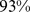 , 2.55	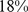 , 3.84	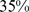 , 4.76	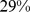 , 4.53

**Table 7:** Convergence time and hit rate results for continuous, FMS and SLE problems through *external* and *internal* adaptive lattice reconfiguration with *anisotropic local selection*.

Adaptive Phenotypic					
*External* Reconfig.	Rastrigin	Griewank	Langerman	FMS	SLE
Rectangular 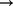 Adapt.	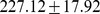	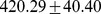	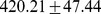	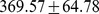	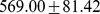
	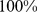	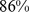 , 3.46	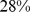 , 4.48	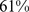 , 4.87	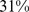 , 4.62
Narrow 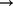 Adapt.	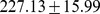	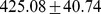	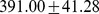	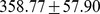	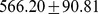
	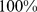	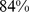 , 3.66	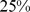 , 4.33	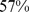 , 4.95	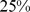 , 4.33
*Internal* Reconfig.					
Square 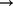 Adapt.	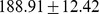	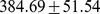	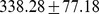	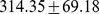	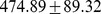
	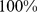	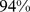 ,2.37	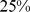 ,4.33	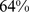 ,4.8	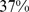 ,4.82
4( 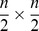 ) 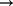 Adapt.	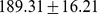	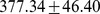	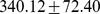	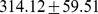	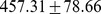
	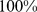	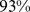 ,2.55	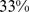 ,4.70	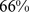 ,4.73	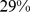 ,4.53

In both tables, statistical difference is proved for most problems except for the SLE problem in Table 7. Therefore, multiple comparison tests were carried out among most approaches. The Rastrigin function shows statistical difference in convergence time for both sets of results. However, no statistical proof supports this difference between best approaches on each table. In comparison to a static square topology, convergence time consistency is held in constant and adaptive criteria.

The Griewank function is better approached by *internal* reconfiguration, square 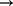 constant for best convergence time, and 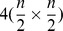 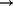 constant for best hit rate. Similar results are obtained through adaptive approaches. Genotypic and phenotypic based *internal* reconfiguration also provides the best performance. In terms of the hit rate, 93% and 94% are reached. However, after multiple comparison tests, no statistical proof was obtained between constant and adaptive criteria. Moreover, consistency in convergence time is kept through *internal* and adaptive *external* mechanisms.

The Langerman function also provides best performance through constant and genotypic *internal* reconfiguration. Similar to the previous function, statistical proof, in convergence time, was not found between the best constant and adaptive approaches. On the other hand, convergence time consistency is slightly lost through *internal* constant and adaptive reconfiguration. In terms of hit rate, square 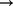 constant and 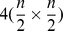 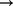 adaptive achieve up to 37% and 38% success rate respectively. This represents a significant improvement in efficacy in comparison to a static square topology, from 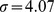 to 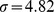 and 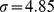 in standard deviations.

The FMS problem is highly epistatic, which means genetic interdependency is strong. Constant *internal* reconfiguration gives the best performance through 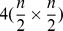 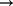 constant mechanism with statistical significant difference. On the other hand, adaptive genotypic *internal* reconfiguration mechanisms provide the best convergence time and hit rate respectively. However, once again no statistical proof was obtained between constant and adaptive reconfiguration techniques. Moreover, best hit rates are 70% and 71% in both cases. Consistency in convergence time is maintained among reconfiguration approaches.

The best convergence time for the SLE problem is achieved through 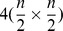 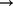 constant *internal* reconfiguration together with the highest hit rate of 42%. Statistical difference is proved in the average number of generations with respect to a static square topology, as well as in hit rates where the difference in standard deviation ranges from 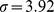 to 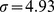. In contrast, results for adaptive approaches are not significantly different in convergence time and a best hit rate of 45% is achieved through genotypic based *internal* reconfiguration. Convergence times are consistent among adaptive mechanisms.

It is worth noticing that through static rectangular topologies most of the problems in Table 6 lose performance quality, increasing convergence time and limiting their hit rates to the minimum, except for the Rastrigin function. Moreover, for static narrow lattices, three of the problems do not converge at all. An important observation is that the best performance results are obtained through constant and adaptive *internal* reconfiguration mechanisms.

In Tables 8 and 9, results for the GPS and combinatorial problems are presented. For most of the problems in each table, statistical difference was found in terms of convergence times, except for the MMDP problem in Table 8.

**Table 8:** Convergence time and hit rate results for GPS and combinatorial problems through static, preprogrammed (*external*) and constant (*internal*) lattice reconfiguration with *anisotropic local selection*.

Static/Preprog.					
*External* Reconfig.	GPS	MMDP	MTTP	P-Peaks	ECC
Square	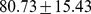	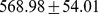	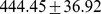	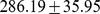	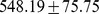
	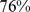 , 4.27	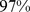 , 1.70	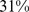 , 4.62	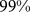	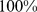
Rectangular	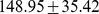	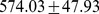	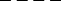	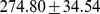	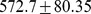
	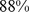 , 3.24	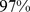 , 1.70	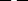	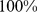	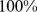
Narrow	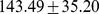	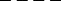	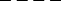	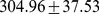	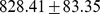
	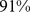 , 2.86	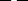	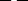	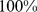	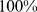
Square-Narrow	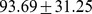	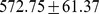	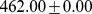	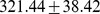	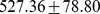
	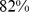 , 3.84	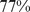 , 4.20	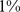 , 0.99	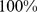	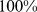
Narrow-Square	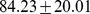	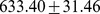	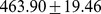	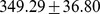	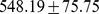
	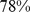 , 4.14	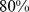 , 4.00	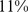 , 3.12	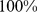	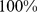

**Table 9:** Convergence time and hit rate results for GPS and combinatorial problems through *external* and *internal* adaptive lattice reconfiguration with *anisotropic local selection*.

Adaptive Phenotypic					
*External* Reconfig.	GPS	MMDP	MTTP	P-Peaks	ECC
Rectangular 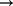 Adapt.	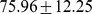	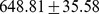	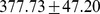	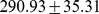	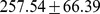
	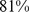 , 3.92	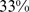 , 4.70	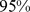 , 2.17	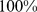	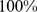
Narrow 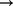 Adapt.	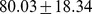	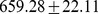	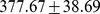	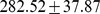	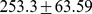
	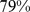 , 4.07	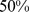 , 5.00	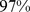 , 1.70	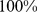	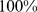
*Internal* Reconfig.					
Square 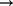 Adapt.	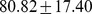	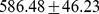	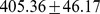	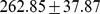	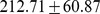
	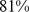 , 3.92	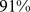 , 2.86	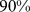 , 3.0	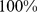 ,	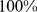 ,
4( 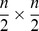 ) 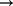 Adapt.	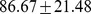	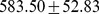	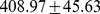	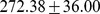	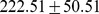
	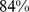 , 3.66	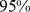 , 2.17	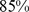 , 3.57	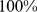 ,	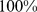 ,

Overall, the GPS problem is the smallest problem. Yet, its landscape is multi-modal and non symmetric also presenting high epistasis. A smaller population size was implemented with 64 individuals and for anisotropic selection, statistical proof was obtained in convergence time. Overall, the best convergence time is achieved through adaptive genotypic based *external* reconfiguration, although a substantially better hit rate of 91% is reached through a static narrow topology. On the other hand, compared with a static square topology, consistency in convergence time is preserved through constant *internal* and adaptive approaches.

The last four problems are combinatorial. Hit rates of 97% are achieved through static square and rectangular topologies for the MMDP problem, while narrow topology cannot converge at all. No statistical proof is obtained in terms of convergence time. For adaptive approaches, the best performance was obtained through phenotypic and genotypic based 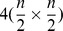 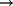 adaptive *internal* reconfiguration with statistical difference in convergence time and 95% success search rate. After the multiple comparison test, statistical proof is also found between best convergence times on each table while consistency is also maintained.

**Table 10:** Convergence times comparison for best *external* and *internal* adaptive approaches versus pre-programmed, static and constant lattice reconfiguration mechanisms through anisotropic local selection.

	Best adaptive approach	*External* static and pre-programmed					*Internal* constant	
		Sqr.	Rect.	Narr.	Sqr.-Narr.	Narr.-Sqr.	Sqr. 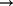 Cte.	4( 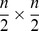 ) 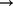 Cte.
Rastrigin	*Internal* Phenotypic Sqr. 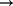 Adapt.	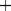	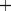	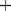	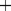	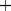	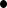	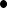
Griewank	*Internal* Genotypic Sqr. 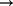 Adapt.	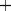	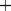	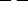	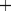	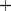	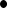	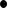
Langerman	*Internal* Genotypic Sqr. 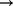 Adapt.	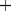	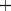	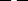	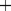	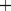	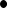	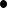
FMS	*Internal* Genotypic 4( 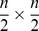 ) 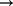 Adapt.	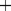	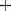	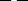	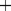	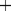	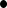	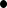
SLE	*Internal* Genotypic 4( 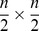 ) 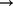 Adapt.	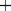	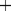	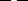	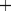	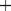	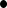	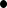
GPS	*External* Genotypic Narr. 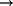 Adapt.	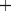	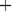	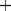	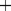	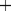	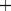	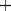
MMDP	*Internal* Genotypic 4( 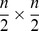 ) 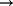 Adapt.	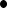	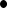	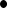	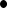	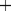	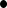	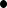
MTTP	*External* Genotypic Narr. 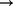 Adapt.	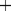	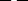	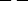	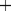	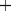	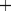	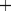
P-Peaks	*External* Genotypic Rect. 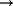 Adapt.	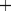	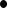	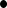	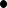	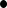	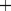	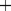
ECC	*External* Genotypic Rect. 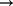 Adapt.	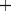	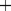	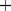	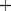	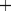	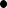	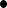

The MTTP presents a very low hit rate for a static square population topology. Moreover, through rectangular and narrow topologies, cGAs are not appropriate to solve this problem. However, the best convergence time and hit rate are achieved by constant *internal* reconfiguration and the hit rate is improved up to 100%. Adaptive approaches show, with statistical difference, improvement in terms of convergence time. Adaptive genotypic based *external* reconfiguration outperforms other approaches. Yet, hit rate reaches 98% and 99% in best cases. Convergence time consistency is kept among *internal* constant and adaptive reconfiguration.

Overall, hit rates for the P-Peaks and the ECC problems are 100%. In terms of convergence time, statistical tests prove an improvement. Constant *internal* and adaptive genotypic based *external* reconfiguration outperform the rest of the proposed techniques, although, there is no statistical proof to differentiate between both approaches. The consistency of the results is well maintained between the best approaches in both problems.

Similar to the results in Table 6 for static, pre-programmed and constant approaches; in Table 8 best convergence times and most of the best success search rates correspond to constant *internal* reconfiguration. Having a static topology or to perform a pre-programmed change in population topology during evolution does not provide the best performance cGAs could achieve. However, in some cases better hit rates are obtained through static and pre-programmed reconfiguration approaches.

Considering take-over time analysis carried out in Section, less influence on cGAs’ performance was expected when lattice reconfiguration mechanisms with binary tournament selection are applied. In contrast, due to a wider span for controlling selective pressure showed by anisotropic selection when 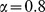, a significant effect in performance was anticipated; as supported by statistical tests carried out for each benchmark problem.

In Table 10, convergence time results for adaptive lattice reconfiguration mechanisms with anisotropic selection are statistically compared. In the second column, the best adaptive approach per problem with statistical significance is included, either best *external* or *internal* lattice reconfiguration criterion. *Internal* adaptive mechanisms outperformed *external* adaptive ones in six out of ten benchmark problems. In columns 3-9, static, pre-programmed and constant reconfiguration mechanisms are statistically compared to best adaptive ones. In most cases, adaptive approaches outperformed static and pre-programmed ones. However, no statistically supported evidence is found for adaptive mechanisms to improve *internal* constant lattice reconfiguration but in three cases (GPS, MTTP and P-Peaks) where genotypic based *external* adaptive approaches outperformed constant ones. Efficacy achieved is very similar when applying adaptive or constant mechanisms with no statistical significant difference. Next section provides final conclusions of this study.

## Discussion

The aim of this research has been to extend previous studies on how structural properties in cGAs can be a factor in adequately tuning diversity during evolution. *Internal* lattice reconfiguration has been experimentally assessed and compared with *external* reconfiguration proposed in [26]. The main difference between both approaches is that individ’uals adjacency is maintained through *internal* lattice reconfiguration while it is lost in the *external* approach because it induces an implicit migration mechanism. Making difficult to distinguish if performance improvements are a consequence of the added effect of the lattice reconfiguration mechanism and the induced migration.

Binary tournament showed a narrow span for modifying selective pressure when *internal* lattice reconfiguration is performed. Thus, a significant effect on selective pressure to tune diversity and consequently in performance was not expected. In contrast, take-over times for anisotropic selection showed a wider span to affect selective pressure when the population’s topology is internally reconfigured; for most of the problems statistical difference was proved in terms of convergence time.

*Internal* adaptive approaches outperformed *external* ones in six out of ten benchmark problems when applying anisotropic local selection. In order to statistically compare the best adaptive criteria, tests were carried out versus static, pre-programmed and constant reconfiguration mechanisms. In three out of ten benchmark problems, adaptive approaches (genotypic based *external* reconfiguration) outperformed constant *internal* reconfiguration.

In cGAs, exploration is carried out globally throughout the grid while exploitation is mostly promoted locally within neighbourhoods. The proposed mechanism for diversity tuning works at a global level through the internal reconfiguration of the population’s topology as a way of modifying the induced selective pressure and thus promote a more balanced exploration-exploitation trade-off. Therefore, its effect does not need to be a prompt response to changes in the solutions or the representation spaces; this is why constant reconfiguration of the topology induces a similar effect in performance as measuring phenotypic or genotypic entropy and is less computationally expensive.
